# Specific Influences of Early Acoustic Environments on Cochlear Hair Cells in Postnatal Mice

**DOI:** 10.1155/2018/5616930

**Published:** 2018-04-16

**Authors:** Aoshuang Chang, Peng Chen, Shasha Guo, Nana Xu, Wenlu Pan, Hongzheng Zhang, Cuixian Li, Jie Tang

**Affiliations:** ^1^Department of Physiology, School of Basic Medical Sciences, Southern Medical University, Guangzhou, China; ^2^School of Basic Medical Sciences, Guizhou Medical University, Guiyang, China; ^3^Department of Otolaryngology Head and Neck Surgery, Zhujiang Hospital, Southern Medical University, Guangzhou, China; ^4^Institute of Mental Health, Southern Medical University, Guangzhou, China

## Abstract

The auditory function develops and matures after birth in many mammalian species. After hearing onset, environmental sounds exert profound and long-term effects on auditory functions. However, the effects of the acoustic environment on the functional development of the peripheral auditory system, especially the cochlear sensory hair cells, are still unclear. In the present study, we exposed mouse pups to frequency-enriched acoustic environments in postnatal days 0–14. The results indicated that the acoustic environment significantly decreased the threshold of the auditory brainstem response in a frequency-specific manner. Compared with controls, no difference was found in the number and alignment of inner and outer hair cells or in the length of hair bundles after acoustic overstimulation. The expression and function of prestin, the motor protein of outer hair cells (OHCs), were specifically increased in OHCs activated by acoustic stimulation at postnatal days 7–11. We analyzed the postnatal maturation of ribbon synapses in the hair cell areas. After acoustic stimulation, the number of ribbon synapses was closer to the mature stage than to the controls. Taken together, these data indicate that early acoustic exposure could promote the functional maturation of cochlear hair cells and the development of hearing.

## 1. Introduction

Acoustic information carried by sound waves is transduced into electric signals by cochlear hair cells (HCs) and transmitted to spiral ganglion neurons (SGNs) and central auditory nuclei. In many mammalian species, auditory function develops and matures after birth. In rodents, the spikes of cortical neurons in response to acoustic stimulation are initially observed after postnatal days 10 to 12 (P10–P12), which is defined as the “hearing onset” [1–3]. During a brief postnatal period around the hearing onset, the structure and function of the cochlea and central auditory system undergo marked changes [3–5]. The auditory system is susceptible to environmental sounds during this short period. Early exposure to an acoustic environment can quickly change the sensitivity and frequency selectivity of neurons at many levels along the auditory pathway [3, 5]. Early exposure to environmental sounds modulates the innervations and response properties of neurons in the auditory pathway [5–9]. It is well recognized that these neuronal mechanisms are involved in the plasticity induced by early exposure to environmental sounds in the auditory system. As all input signals for neural activity originated from HCs, which are the peripheral sensory cells of the auditory system, any inference on HCs may have a major effect on the development of the auditory function. However, little is known about whether early experience influences the development of cochlear HCs.

The maturation of the cochlea, which is the peripheral auditory system, is also completed after birth. The mechanoelectrical transduction (MET), electromotility, and synapse transmission are fundamental functions of HCs and are critical for normal hearing [10]. In murines, the pattern of hair bundles is formed before postnatal day 6 [11–14]. The functional development of MET is completed at the same age [4, 11]. The electromotility of outer hair cells (OHCs) are first detected at P6 and are gradually developed after that [15, 16]. Meanwhile, the maturation of both efferent and afferent innervations is also established after birth [17]. These findings imply that the HCs have the capability of responding to sound signals at the early postnatal stage. We are curious about whether the sound environment could elicit any morphological or functional changes of hair cells during this period.

In the present study, we aimed to clarify whether the environmental sound can regulate the developmental processes of these HC functions. We applied sound exposure at different frequencies to stimulate the mouse pups starting at P0–P14 and found that the auditory brainstem responses (ABRs) were enhanced by acoustic stimulation in a frequency-specific manner. No change was observed in the development of stereocilia in the HCs. The sound enhanced the expression and function of prestin, a motor protein on the outer hair cells, and the refinement of ribbon synapses of the inner hair cells (IHCs). Our results suggest that early exposure to acoustic environments could promote the functional maturation of cochlear HCs and the development of hearing.

## 2. Material and Methods

All experiments were performed in accordance with the Chinese Prevention of Cruelty to Animals Act, and approval was obtained from the Southern Medical University Laboratory Animal Center.

### 2.1. Animal Care and Early Acoustic Stimulation

Adult C57BL/6 mice of equal sex and pups ranging in age from P0 to P14 were used in this study. The control animals were housed in a normal sound environment (with environmental sound level of 40–50 dB sound pressure level (SPL)) under a 12 h light/dark schedule and had free access to water and a standard diet. For the treated group, litters of P0 and their mothers were housed in a soundproof chamber for 14 days. A 12 h light/dark schedule was established in the soundproof chamber. For each litter, one of three different acoustic environments was used to stimulate the mouse pups: narrow band (0–2 kHz) noise burst, 16 kHz, and 32 kHz tone bursts. The sounds were delivered by a loudspeaker (ES1, Tucker-Davis Technologies, Alachua, FL, USA) placed on the ceiling of the chamber, ~40 cm away from the animals. The mouse litters were subjected to an alternation of 250 ms long noise or tone bursts and 250 ms quiet intervals 24 h/day for 14 days at a sound level of 70 dB SPL. The stimulus and environmental sound levels were calibrated by a B&K measuring amplifier through a B&K 4135 microphone placed in the center of the chamber. No distortion or significant harmonic signal was found in the chamber when a stimulus was delivered. Compared with the control group, no obvious weight loss was observed in the sound-stimulated litters, indicating normal lactation.

### 2.2. Auditory Brainstem Response (ABR) Recording

Animals were anesthetized with an intraperitoneal injection of sodium pentobarbital (22 mg/kg for pups and 30 mg/kg for adults) and subsequently placed on an antivibration table in a soundproof room. During ABR recording, a heating pad was used to maintain the animal's body temperature at 37.5°C. By using fine scissors, a 1-2 mm incision ventroposterior to the external pinna was made to place the indifference or ground electrode. A subdermal needle electrode (recording electrode) was located over the skull vertex. Tone bursts (1 ms rise/fall, 3 ms plateau) of various frequencies (1, 2, 4, 8, 16, 24, and 32 kHz) and intensities (0–90 dB SPL at 5 dB intervals) were delivered using a loudspeaker (ES1, Tucker-Davis Technologies, Alachua, FL, USA) placed 10 cm in front of the animal. The sound levels were calibrated by a B&K measuring amplifier through a B&K 4135 microphone placed 10 cm in front of the speaker. The tone bursts with different frequency and amplitude were controlled by TDT System 3 (Tucker-Davis Technologies, Alachua, FL, USA) and were delivered in a randomized sequence. Each frequency-amplitude combination was repeated 256 times at a rate of 10 bursts/s. The ABR signals were amplified, filtered (100–1000 Hz), averaged using TDT System 3, and recorded using BioSigRP software (Tucker-Davis Technologies, Alachua, FL, USA). Hearing thresholds were defined as the minimum sound intensity at which averaged waveform II could be detected. Data were stored for offline analysis. The entire recording for one animal spanned over approximately 60 min.

### 2.3. Tissue Dissection and Immunostaining

After physiological experiments, the mouse was sacrificed using CO_2_ and the skull was opened along the sagittal midline. The brain was removed and the inner ears were isolated from the temporal bones and transferred into a Petri dish filled with ice-cold L-15 medium (Invitrogen, Carlsbad, CA, USA). Under the dissection microscope, the bony capsule of the cochlea was removed. The organ of Corti and associated stria vascularis were unwrapped from the modiolus. By holding the basal portion of the stria vascularis, the organ of Corti was separated from the stria vascularis completely by unwinding slowly from base-to-apex. The apical, middle, and basal turns of the organ of Corti were cut by using fine scissors. The segments of the organ of Corti were fixed in 4% paraformaldehyde for 4 h and decalcified in 6% ethylenediaminetetraacetic acid (EDTA) for 3 additional days. Tissues were blocked with 10% goat serum at 27°C for 30 min after permeabilization using 0.3% Triton X-100 in phosphate-buffered saline (PBS) for 30 min. After incubation with anti-prestin antibody (1 : 100, Santa Cruz Biotech, Santa Cruz, CA, USA) or anti-CtBP2 antibody (1 : 500, Proteintech, Chicago, IL, USA) overnight at 4°C, the tissues were subsequently incubated with Alexa Fluor 488-conjugated Goat Anti-rabbit IgG (1 : 600, Invitrogen, Carlsbad, CA, USA) at 27°C for 1 h. After washing with PBS, rhodamine fluorescence phalloidin (1 : 200, Sigma, St. Louis, MO, USA) was used to label the hair bundles. The segments were mounted with ProLong Gold antifade Mountant reagent (Invitrogen, Carlsbad, CA, USA) on a glass slide. The fluorescence images were acquired using a confocal microscope (A1+, Nikon, Japan). To quantify ribbon synapses, fluorescence images were acquired using z-scan, with a step of 0.8–1.2 *μ*m between each plane.

### 2.4. Scanning Electron Microscopy (SEM)

The cochleae of P3–P14 animals were collected after ABR measurements and were fixed for 2 h in glutaraldehyde (3% in PBS). The round window and apex of the cochlea were opened to provide access to the fixative. After decalcification in 10% EDTA for 2 d, the specimens were rinsed with PBS and fixed again in osmium tetroxide (1% in PBS) for 1 h at 27°C. The bony wall of the cochlea was removed to expose the organ of Corti. Subsequently, gradient alcohol was used for dehydration (15 min). After critical point drying and coating with gold, the specimens were imaged by using SEM (H-3400, Hitachi, Tokyo, Japan). The length of the OHC and IHC stereocilia was measured at different postnatal ages.

### 2.5. Western Blotting

The segments of the dissected organs of Corti were collected and homogenized with ultrasonic cell crushers (VCX150, Sonic, Newtown, CT, USA). The supernatants of the homogenates were subjected to 8% sodium dodecyl sulfate polyacrylamide gel electrophoresis and transferred to nitrocellulose blotting membranes (Millipore, Bedford, MA, USA). After blocking with 5% nonfat milk in TBS-T (20 mM Tris, 137 mM NaCl, and 0.1% Tween 20, pH 7.6) for 1 h at 27°C, the membranes were incubated with anti-prestin or anti-GAPDH antibodies overnight at 4°C, followed by horseradish peroxidase-conjugated secondary antibodies for 1 h at 27°C. The immunobands were detected by a chemiluminescence detection system (GE Healthcare, Chicago, IL, USA) and visualized using the Amersham Imager 600 (AI600, GE Healthcare, Chicago, IL, USA). The intensity of the immunobands was quantified using MultiGauge software (GE Healthcare, Chicago, IL, USA). Data were obtained from four independent experiments.

### 2.6. Quantitative Polymerase Chain Reaction (qPCR) Assay

After ABR measurements, the apical segments of the cochlear basal membrane were quickly peeled and total RNA was extracted using TRIzol reagent (Invitrogen, Carlsbad, CA, USA). The purity and completeness of the extracted RNA were evaluated to obtain high-quality RNA. Subsequently, cDNA was synthesized with the PrimeScript RT master mix kit (RRO36A, Takara). The reverse transcription (RT)-qPCR assay of prestin was carried out using the ABI Real-Time PCR system (StepOnePlus, Thermo Fisher Scientific, Waltham, MA, USA); GAPDH was used as the internal reference. Two pairs of primers (Invitrogen, Carlsbad, CA, USA) were used [18]: Prestin, forward: 52032-CGACTTGTATAGCAGCGCTTTAAA -3′, reverse: 5′-TTCTTCTCGCTCCCATAATGAGT-3′; GAPDH, forward: 5′-CTTCGATGCCGGGGCTGGCATT-3′, reverse: 5′-TGTTGGGGGCCGAGTTGGGATAGG-3′. Data were obtained from four independent experiments.

### 2.7. Nonlinear Capacitance (NLC) Measurement of OHCs

The NLC recording of isolated OHCs was performed as described in our recent study [19]. The dissected segments of the organ of Corti were digested using collagenase IV for 5 min (2 mg/ml, Sigma, St. Louis, MO, USA). After gentle pipetting, the OHCs were isolated from the organ of Corti and were transferred into a small plastic chamber filled with enzyme-free L-15 medium (pH 7.35 and 300 mOsm). Healthy looking OHCs were selected for whole-cell patch clamp recording. The recording electrode was pulled using a pipette puller (P97, Sutter, Sacramento, CA, USA) and filled with intracellular solution (120 mM CsCl, 2 mM MgCl_2_, 10 mM EGTA, and 10 mM HEPES, 300 mOsm, pH 7.3). Typically, the impedance of patch pipette was 5–8 MΩ in bath solution (L-15). Classical whole-cell patch clamp recording was performed using an Axopatch 700B amplifier and a 1440 A/D converter (Molecular Devices, San Jose, CA, USA). A two sine-wave voltage stimulus protocol was generated by using jClamp 32 (Scisoft, CT, USA) to measure the membrane capacitance of OHCs at different membrane voltages. Data were stored and analyzed by the jClamp software offline. The capacitance can be fitted with a two-state Boltzmann function and can reflect the nonlinear charge movements to membrane voltage [20]. The Boltzmann function for capacitance fitting is calculated as follows:

(1) $$\begin{array}{c} C_{m}=C_{lin}+\frac{Q_{\max}\alpha }{\exp\left[\alpha \left(V_{m}-V_{1/2}\right)\right]{\left(1+\exp\left[-\alpha \left(V_{m}-V_{1/2}\right)\right]\right)}^{2}}, \end{array}$$

where *C*_*m*_ is the membrane capacitance, *C*_lin_ is the linear capacitance which is related to the surface area of the cell membrane, *Q*_max_ the maximum charge transfer across the membrane, *V*_1/2_ (or *V_h_*) is the membrane voltage at which the maximum charge movement occurs, or, equivalently, the peak of the capacitance-voltage function, and *α* represents the slope of the voltage dependence (*α* = *ze*/*kT*, where *k* is the Boltzmann constant, *T* is the absolute temperature, *z* is the valence of the charge movement, and *e* is the electron charge). To compare the magnitude values of the NLC obtained from OHCs with various cell sizes, we normalized NLC and *Q*_max_ and *C*_lin_.

### 2.8. Statistical Analysis

Excel and SPSS software were used for the calculations, analysis, and plotting. The *t*-test was used to examine the significance between different data groups. The significance between different NLC responses was examined by repeated ANOVA, mixed linear model. Significance was defined as *p* < 0.05.

## 3. Results

**Figure 1:**
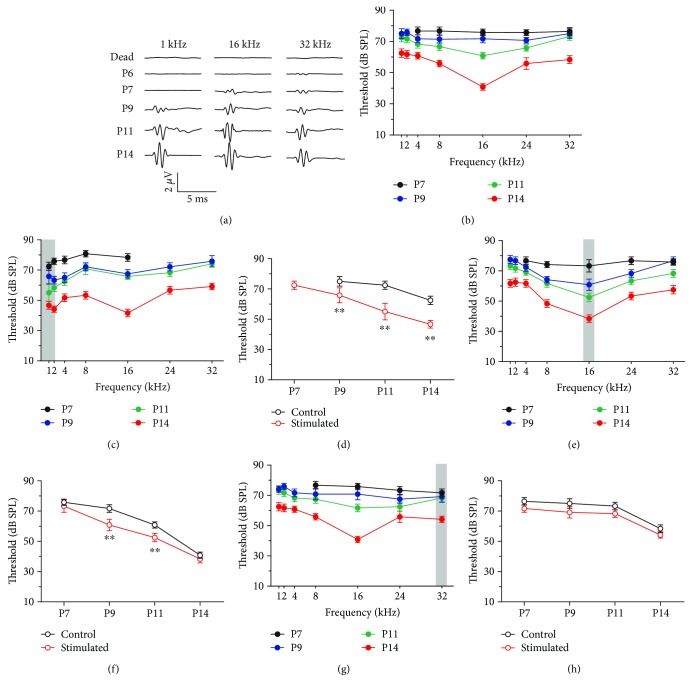
Effects of acoustic stimulation on ABR responses at different postnatal ages. (a) Representative ABR waveforms in response to 1 kHz, 16 kHz, and 32 kHz tone bursts (80 dB SPL) at different postnatal ages. The traces recorded from dead animals show that no stimulation artifact was observed. (b) The ABR threshold frequency-tuning curves at different postnatal ages (P7, *N* = 7; P9, *N* = 7; P11, *N* = 6; P14, *N* = 6). The color code in (b) applies for (c), (e), and (g). (c) The ABR threshold frequency-tuning curves at different postnatal ages after low-frequency sound environment stimulation. The grey area indicates the stimulation frequency used for the sound environment (P7, *N* = 6; P9, *N* = 7; P11, *N* = 6; P14, *N* = 6). (d) The changes in ABR threshold in response to a 1 kHz tone burst during postnatal development for those animals in (c) (red) and control animals in (b) (black). (e) The ABR threshold frequency-tuning curves at different postnatal ages after 16 kHz sound environment stimulation (P7, *N* = 6; P9, *N* = 6; P11, *N* = 6; P14, *N* = 6). (f) The changes in ABR threshold in response to a 16 kHz tone burst during postnatal development for those animals in (e) (red) and control animals in (b) (black). (g) The ABR threshold frequency-tuning curves at different postnatal ages after 32 kHz sound environment stimulation (P7, *N* = 6; P9, *N* = 6; P11, *N* = 6; P14, *N* = 6). (h) The changes in ABR threshold in response to a 32 kHz tone burst during postnatal development for those animals in (g) (red) and control animals in (b) (black). All data points represent the mean ± standard deviation. ^∗∗^*p* < 0.01. ABR: auditory brainstem response; *N*: number; P: postnatal day.

The ABR was examined first in postnatal mice in the control group. During the first few days after birth, no ABR was detectable until P7. At P7, the ABR was elicited only by high-frequency tone bursts at high sound level, whereas no ABR was detectable for mid- and low-frequency tone bursts (Figure 1(a)). In the following days, the frequency range of ABR extended gradually from high frequency to low frequency. At P11, robust ABRs could be measured in response to all tested frequencies. At P14, the ABR amplitudes were comparable with those of adult animals. As shown in Figure 1(b), the ABR thresholds decreased with postnatal age, indicating that auditory function of mice pups developed between P7 and P14. Hearing in mice started from a high-frequency range and extended later to low frequency. In the present study, we raised mice in controlled-sound environments. Low-frequency noise (0–2 kHz) was used to stimulate the mice pups (gray area in Figure 1(c)); the frequency threshold-tuning curve was measured based on recorded ABR data. Compared with the control group, the ABR thresholds decreased significantly in response to the stimulus frequency (1 kHz). Moreover, the first day at which the ABR in response to 1 kHz tones appeared preceded from P9 to P7 (Figure 1(d)). Similar effects on ABR development by 16 kHz and 32 kHz acoustic environments were observed, as shown in Figures 1(e)–1(h). However, these influences were only observed in mice pups younger than P14. The acoustic environments have no effect on the ABR thresholds of adult animals (i.e., the mothers; data not shown). These results suggest that pups were more sensitive to low-frequency sound environments. Therefore, the effects of low-frequency sounds were investigated. Consistent with our previous study [21], waves IV to VII were absent in the ABR waveforms recorded from pups in the present study. This observation may reflect the immature function of the central auditory system. These data suggest that the sound environment changed the function of the peripheral auditory system during early development. Since the responses of cochlear HCs and spiral ganglion neurons contribute greatly to the ABR waves I and II [10], we further examined the impact of the environment enriched by low-frequency noise burst on the morphology and function of HCs.

**Figure 2:**
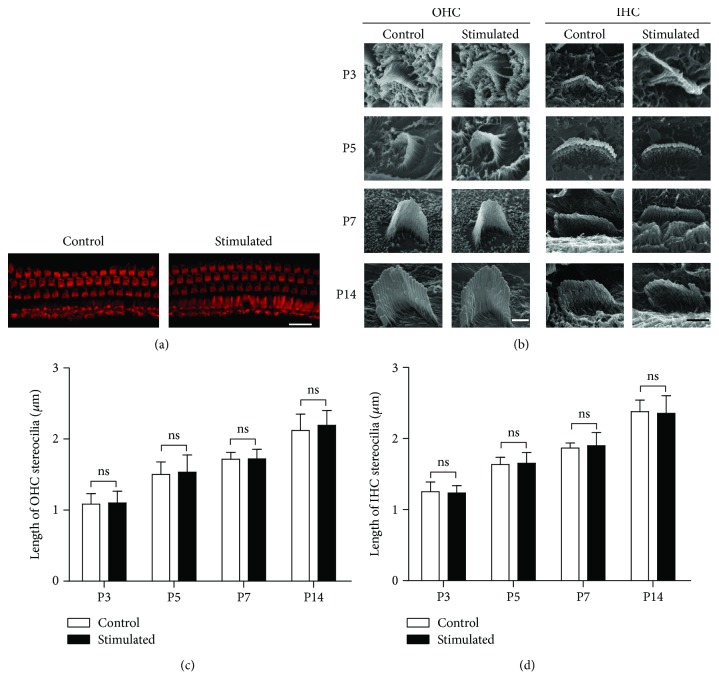
Low-frequency sound environment did not change the alignment and the growth of hair bundles in hair cells in the apical turn of the cochleae. (a) Three rows of OHCs and one row of IHCs (labeled with rhodamine-phalloidin (red)) were observed in both acoustic-stimulated and control groups at P14. Scale bar = 20 *μ*m. (b) Scanning electron microscope images of the hair bundles of OHCs and IHCs in the apical turns of the cochleae at different postnatal ages. Scale bar = 1 *μ*m for OHCs. Scale bar = 2 *μ*m for IHCs. (c) and (d) depict the length of OHC (c) and IHC (d) stereocilia at different postnatal ages. At least six cells from six cochleae were measured for each column. All data points represent as mean ± standard deviation. INC: inner hair cell; ns: no significance; OHC: outer hair cell; P: postnatal day.

**Figure 3:**
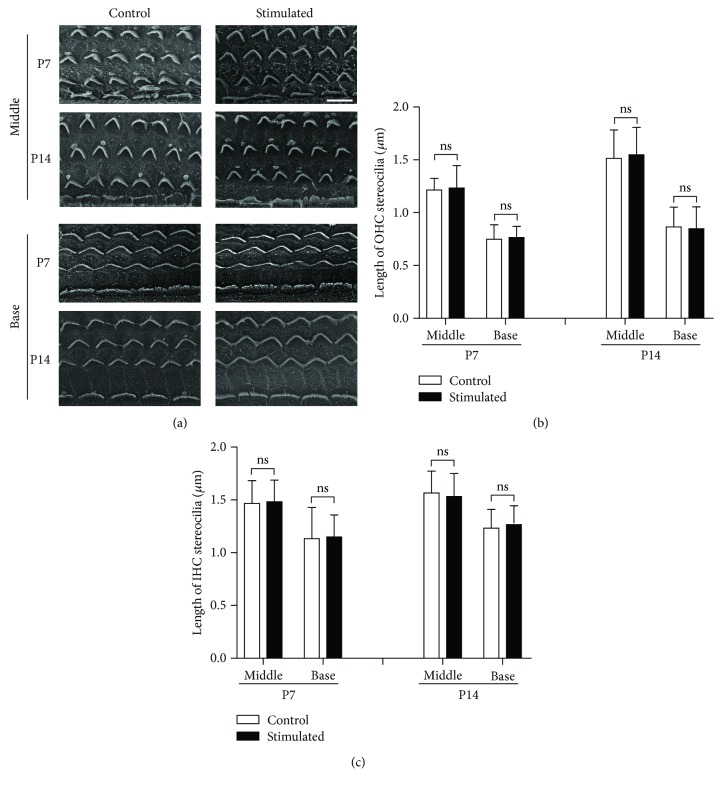
Low-frequency sound environment did not change the alignment and the growth of hair bundles in hair cells in the middle and basal turns. (a) Scanning electron microscope images show the hair bundles of OHCs and IHCs in the middle and basal turns at different postnatal ages. Scale bar = 10 *μ*m. (b and c) The length of OHC (b) and IHC (c) stereocilia at different postnatal ages, compared between low-frequency sound environment-stimulated and control groups. At least six cells from six cochleae were measured for each column. All data points represent the mean ± standard deviation. INC: inner hair cell; ns: no significance; OHC: outer hair cell; P: postnatal day.

Figure 2(a) depicts the HCs located in the apical segment of the basilar membrane of the cochlea, the segment corresponding to low frequencies. The OHCs were clearly lined up in three rows and IHCs in one row in both the control group and the group treated with 14-day exposure to low-frequency sounds (Figure 2(a)). No observable difference was identified between the two groups. Further examination of the OHC/IHC hair bundles was made based on SEM images as shown in Figure 2(b). Compared with the control group, no significant change in the length of stereocilia was observed after early sound stimulation both in OHCs and IHCs (Figures 2(c) and 2(d)). For mice raised in low-frequency sound environments, the HCs in the middle and basal locations of the cochlea received less sound stimulation than those HCs in the control group. Their morphology and development were also examined (Figure 3(a)). Together with the measurements of the stereocilia length (Figures 3(b) and 3(c)), our data indicated that low-frequency sound environments have no effect on the growth of HC stereocilia during development, regardless of the location of HCs in the cochlea.

**Figure 4:**
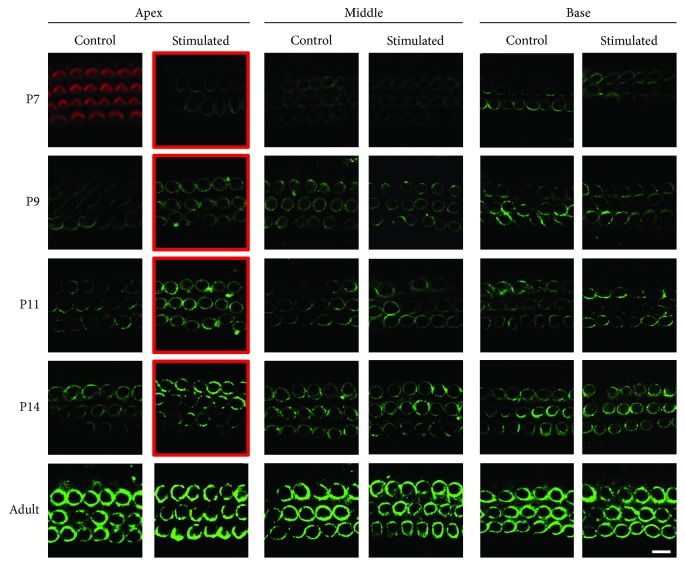
Confocal images indicate that a low-frequency sound environment increases the expression of prestin in apical OHCs during postnatal development. Prestin (green) was labeled in three segments (apex, middle, and base) along the cochlea in P7, P9, P11, P14, and adult mice. For P7 apical turns, no green fluorescence was detected. The hair bundles of hair cells were labeled with rhodamine-phalloidin (red) to indicate the location of the hair cells. The red borders mark the postnatal ages at which prestin expression level was higher in the sound-stimulated group. Scale bar = 10 *μ*m. OHC: outer hair cell; P: postnatal day.

**Figure 5:**
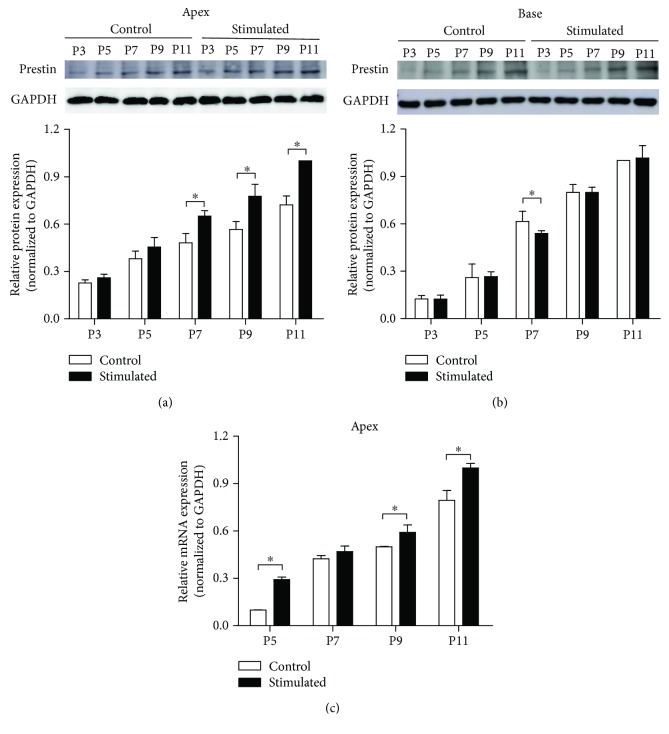
Low-frequency sound environment enhances the expression of prestin in OHCs in the apical turns. (a) Prestin protein expression is increased in the apical organ of Corti after low-frequency sound environment stimulation. Upper panel: immunobands of prestin from the apical organ of Corti at different postnatal ages. Lower panel: normalized prestin expression level. (b) Prestin protein expression is unchanged in the basal organ of Corti after sound environment stimulation. Upper panel: immunobands of prestin at different postnatal ages. Lower panel: normalized prestin expression level. (c) Normalized prestin mRNA level is increased in the apical organ of Corti after sound stimulation. All data points represent the mean ± standard deviation. ^∗^*p* < 0.05. OHC: outer hair cell.

Both IHCs and OHCs convert sound vibration into electrical signals. Moreover, OHCs generate motion by changing their cell length with acoustic stimulation [22]. This motion was driven by prestin, the motor protein on the lateral membrane of OHCs. The immunostaining results showed that the expression of prestin starts in the control group at P7 in OHCs of the basal cochlea (Figure 4). No prestin-related fluorescence was detected before P9 in the OHCs of the apical cochlea. Under a low-frequency sound environment, detectable prestin fluorescence was observed at P7 in the apical OHCs. Furthermore, compared with the control group at the same postnatal ages, the prestin fluorescence was more pronounced from P7 to P14. To evaluate the expression level of prestin, Western blot and qPCR assays were performed in the apical and basal in the organ of Corti. As shown in Figure 5(a), in the apical turn of the cochlea, the expression level of prestin increased with the postnatal ages. Under a low-frequency sound environment, the expression level of prestin was significantly increased from P7 to P11. However, no difference in the prestin level was found in the basal turn of the cochlea, except a slight decrease at P7 (Figure 5(b)). The increase of prestin expression in the apical cochlea was further confirmed by the qPCR results. The prestin mRNA levels were significantly increased by low-frequency sound exposure from P5 to P11 (Figure 5(c)).

**Figure 6:**
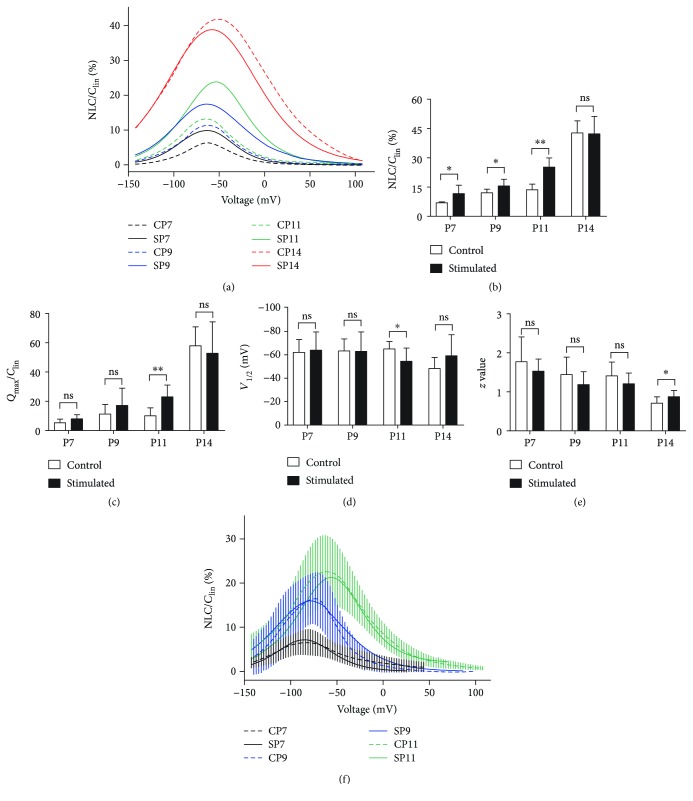
Low-frequency sound environment stimulation enhances the NLC response of OHCs from the apical turns of the cochleae. (a) Means of NLC from low-frequency noise-stimulated (SP, solid lines) and control (CP, dashed lines) OHCs in apical cochlea at P7 (SP7, *n* = 8; CP7, *n* = 5), P9 (SP9, *n* = 9; CP9, *n* = 8), P11 (SP11, *n* = 12; CP11, *n* = 9), and P14 (SP14, *n* = 13; CP14, *n* = 10). The mean capacitance-voltage responses were fitted with the Boltzmann function. (b–e) Four parameters obtained from curve fitting using the Boltzmann function. All data points represent the mean ± standard deviation. ^∗^*p* < 0.05; ^∗∗^ *p* < 0.01; NLC: nonlinear capacitance; ns: no significance; OHC: outer hair cell; P: postnatal day. (f) Means of NLC from low-frequency noise-stimulated (SP, solid lines) and control (CP, dashed lines) OHCs in basal cochlea at P7 (SP7, *N* = 5; CP7, *N* = 12), P9 (SP9, *N* = 4; CP9, *N* = 9), and P11 (SP11, *N* = 4; CP11, *N* = 8). The mean capacitance-voltage responses were fitted with the Boltzmann function. The SDs of mean NLCs are indicated by the vertical bars for the controls. No significant difference was found between the two groups (*p* > 0.05, repeated ANOVA).

We also applied whole-cell patch clamp technology to determine the function of prestin expressed on the OHCs. In OHCs, NLC and electromotility are typically coupled [20, 23]. The NLC can be easily measured and precisely reflects the electrophysiological function of prestin. Therefore, NLC was first measured in OHCs isolated from apical cochlea at different postnatal ages.  Figure 6(a) depicts the NLCs of apical OHCs at different postnatal ages. No NLC was found in response to the changes of membrane voltage before P7. Subsequently, the NLC membrane potential curves changed with the postnatal ages. Low-frequency sound environment increased the capacitance of OHCs compared with the control group. The NLC amplitude (NLC/*C*_lin_) and the moving charge density (*Q*_max_/*C*_lin_) were increased by sound stimulation before P14, while the peak of the capacitance-voltage function (*V*_1/2_) and the shape of the NLC curves (reflected by the *z* value) remained largely unchanged (Figures 6(b)–6(e)). NLC was then measured in OHCs isolated from the basal cochlea. As shown in Figure 6(f), no significant difference was observed between control and low-frequency-exposed groups (*p* > 0.05, repeated ANOVA test).

**Figure 7:**
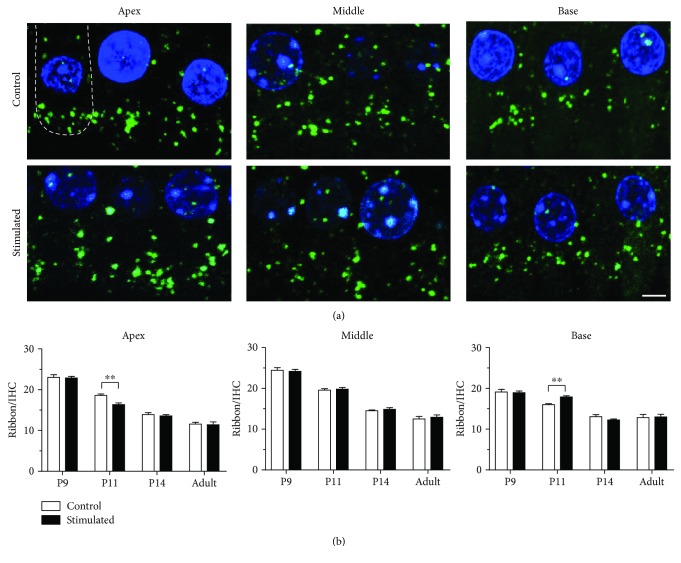
Low-frequency sound environment stimulation influences the changes in ribbon synapses during postnatal development. (a) Representative maximum projections of confocal Z stacks of IHCs in the apical, middle, and basal turns of the cochleae at P11. Anti-CtBP2 and DAPI were used to visualize the IHCs ribbons (green) and nuclei (blue), respectively. The white line in the first image indicates the bottom of an IHC. Scale bar = 5 *μ*m. (b) The number of ribbon synapses per IHC from different locations along the cochlea at different postnatal ages. At least 15 IHCs from 3–5 cochleae were measured for each column. All data points represent the mean ± standard error of the mean. ^∗∗^*p* < 0.01. DAPI: 4′,6-diamidino-2-phenylindole; IHC: inner hair cell; P: postnatal day.

The sound elicited by the vibration of the basilar membrane is amplified by the OHCs and was converted into electrical signals in the IHCs. These signals are transferred to SGNs via ribbon synapses, which are the afferent synapses between IHCs and SGNs. To analyze the postnatal maturation of afferent synapses in the IHC areas, we examined the number of ribbon synapses by evaluating CtBP2, a specific ribbon synaptic marker, in the cochlear sensory epithelium at P9, P11, and P14 (Figure 7(a)). Consistent with previous studies [17, 24, 25], the number of ribbon synapses in IHCs decreased during postnatal development, indicating the refinement of the connections between IHCs and SGNs. As shown in Figure 7(b), the number of CtBP2 clusters per IHC (mean ± standard error of the mean) decreased from 22.2 ± 1.4 at P9 to 13.9 ± 1.4 (mean ± SEM) at P14 in the apical region. Under the administration of low-frequency sound stimulation, the number of CtBP2 clusters per IHC changed from 18.7 ± 1.3 in the controls to 16.4 ± 1.6 at P11 (*p* < 0.05, *t*-test). In the middle region of the cochlea, early sound exposure resulted in no change in the number of CtBP2 clusters. In contrast, in the basal region of the cochlea, low-frequency sound stimulation increased the number of CtBP2 clusters per IHC from 16.0 ± 0.9 in the controls to 17.9 ± 1.1 at P11 (*p* < 0.05, *t*-test).

## 4. Discussion

Life experience and environmental factors markedly affect animal perception, including the hearing function. Although plasticity could be observed throughout the lifespan of animals, this experience-dependent plasticity is extreme during development. Sound stimulation in early life can change the sensitivity and frequency selectivity of the animal's auditory system, by shaping the architecture of the auditory system and modulating the maturation of the auditory function. Many studies have demonstrated that the sound stimulation modulated the innervations of auditory neurons and refined the synaptic functions in the auditory cortex [1–3, 5]. For example, the balance between the excitatory and inhibitory inputs of primary auditory cortical neurons is facilitated by sound stimulation applied to rats younger than P20 [5, 8]. There is no doubt that the plasticity of the auditory central nervous system contributes greatly to the profound and persistent adaption to the sound environment during a brief postnatal epoch. However, the influence of early sound stimulation on the development of the peripheral auditory system has not been elucidated. In the present study, the ABRs of mice pups were examined to determine whether the early exposure to a sound environment could modify the function of the cochlea. In adult mice, ABR usually exhibits 5 to7 peaks generated by distinct nuclei along the ascending pathway of the auditory system. As reported in our previous study, only waves I to III of the ABR waveforms were observed in mice pups before P14 [21]. The absence of later ABR waveforms implies the weak activity of higher stages of auditory nuclei in the early postnatal epoch. The continuous improvement of ABR waveforms and thresholds after hearing onset may imply the maturation of the central auditory system [26]. It is generally accepted that waves I and II of ABR waveforms represent the response of cochlear sensory cells and the auditory nerve [10]. We found that sound stimulation specifically changed the ABR threshold in a frequency-dependent manner, indicating that sound can modulate the function of the cochlear sensory cells (Figure 1).

The morphological and functional maturation of HCs occurs after birth in rodents. The maturation of hair bundles, MET function, electromotility of OHCs, and ribbon synapses is not completed until P10–P14, which is defined as the “onset” of hearing [4, 27, 28]. Any change in these items may greatly influence the function of HCs, resulting in changes in the maturation of the auditory functions. In the present study, we first examined the morphology of hair bundles under the sound environment. The sound-induced deflection of stereocilia in the hair bundles opens the MET channels located on the top of the stereocilia. Normal length and arrangement of stereocilia are required to generate normal MET currents and convert sound waves to electrical signals. Our results indicated that early exposure to the enriched acoustic environment does not change the features of hair bundles during development, including the shape, length, and the arrangement of stereocilia neither in IHCs nor OHCs (Figures 2 and 3). Although we did not measure the MET currents directly, our results suggested that the hair bundle functions may remain unchanged by early sound exposure.

The mammalian OHCs can change the length of their cell body at the frequency of sound signals. This process is termed electromotility and is powered by prestin, the motor protein expressed on the membrane surface of OHCs [29, 30]. Driven by the receptor potential, intracellular chloride moves in and out of prestin and triggers prestin to switch between a long and short conformation, resulting in the elongation and shortening of OHCs [22, 29]. The expression of prestin starts in the OHCs at the basal cochlea at approximately P5 and spreads to apical OHCs in the following days [21, 31]. We found that low-frequency sound environment specifically increased the expression of prestin in apical OHCs, as evidenced by the Western blotting and qPCR data (Figures 4 and 5). Furthermore, the NLC of apical OHCs was also potentiated by low-frequency sound environment at P7–P11, indicating that the electromotility of these OHCs was enhanced (Figure 6). However, such effects could not be obtained in the basal OHCs, although a slightly decreased prestin expression was observed at P7 (Figures 4–6). The changes in the expression and function of prestin can increase the sensitivity to low-frequency sounds and may be, at least partially, responsible for augmented ABR in response to low-frequency sound. However, these changes could only be observed in the period between P7 and P14. No difference was observed in mice older than P14. Therefore, the early exposure to sound can only accelerate the expression of prestin during development but cannot induce the overexpression of prestin in OHCs. It has been reported that the prestin expression level and function were enhanced in residual noise-exposed OHCs. One possible modulating source is the central feedback. The activity of OHCs is modulated by an efferent pathway through an acetylcholine alpha-9/10 receptor. It is not likely that early sound exposure modulates the expression of prestin through this mechanism, because the efferent pathway is not well developed before the onset of hearing [32–34]. The prestin level can be regulated by the thyroid hormone, retinoid nuclear transcription factor, GATA-3, and Pou4f3 during development [35–37]. Further studies are still needed to determine the exact mechanism underlying the impact of early sound exposure on prestin expression in OHCs.

The HC responses are transmitted to SGNs through glutamatergic synapses, which are known as ribbon synapses. Proper number and size of ribbon synapses at the bottom region of HC are critical for the effective transmission of electrical signals from IHCs to SGNs [24, 32, 33]. During the early developmental epoch, the number of ribbon synapses decreased with postnatal age in IHCs at all locations along the cochlea (Figure 7). This result is consistent with previous findings [17, 24, 25]. We found that low-frequency sound environment selectively decreased ribbon synapses in apical IHCs and increased ribbon synapses in basal IHCs, indicating that the neural transmission was also influenced by early sound exposure (Figure 7). Similar to the changes in the expression of prestin, these changes could not be observed after P14.

Although the functional maturation of cochlea occurs considerably faster than that for more central parts of the auditory system, immaturity of conductive factors may limit the efficiency of cochlear responses. The ear canal is closed and the ossicular ossification is incomplete before the hearing onset in rodents [38]. One may raise the question about how sound is transmitted to the cochlea and influence the development of hair cells. The air-filled middle ear cavity and clearance of the auditory canal are not ready for air-0conducted stimulation in the first two weeks after birth. However, ABR to bone-conducted auditory stimuli could be recorded from neonatal rats at age of P7 [38]. We propose that the over sound exposure in the present study is transmitted to the cochlea via the bone conduction and influence the sensorineural development before the hearing onset. This suggestion is supported by the results that high-intensity sound stimuli evoke ABR and behavior response before hearing onset [21, 39]. In our study, low-frequency sound exerted stronger effects on the maturation of HCs than high-frequency sound. This may have two possible explanations. First, the maturation of the basal HCs, which is responsive to high-frequency sound stimuli, occurs earlier than the maturation of apical HCs, which is responsive to low-frequency sound stimuli. This difference in the timing of development allows a longer period for low-frequency sound environments to induce more profound effects on apical HCs. Second, in contrast to the adult middle ear, which is filled with air, the middle ear of pups younger than P11 is filled with liquid. As more energy is attenuated by the liquid and soft ossicular chain in the case of high-frequency sounds, than the low-frequency sound, the latter is more effectively conducted to the inner ear.

Our study suggested that early exposure to sound can enhance the function of HCs and accelerate the development of the peripheral auditory system. However, early exposure to sound environment induced effects on the cochlea that were different from the plasticity in the central auditory nervous system during development. The changes in HCs observed in the present study are not persistent. After P14, most HCs features showed no differences between the control- and sound-exposed groups, implying that peripheral HC development is largely controlled by intrinsic genetic makeup. The early exposure to sound accelerates the development of HCs by so far unknown factors. Compared with the control group, pups exposed to low-frequency sound received less stimulation at high frequency. In addition, the development of HCs in the basal cochlea was delayed under the exposure to the low-frequency sound environment as evidenced by the Western blotting and ribbon synapse staining results (Figures 5 and 7). These results suggest that sound plays a role in modulating the development of inner ear sensory cells. However, in the present study, the effect of the sound environment on HCs occurred before the hearing onset (approximately at P10). This implies that the development of the central neural system requires matured HCs to provide proper input signals. Thus, although the modulation in HCs is not as profound as the plasticity in central neurons during development, the effects of early sound exposure on HC functions may result in persistent changes in the whole auditory system by altering the input signals. Our findings provide a new strategy to modulate the function of cochlear sensory cells during development. This approach is safer and easier to apply than drug and gene therapies.

## 5. Conclusions

In conclusion, our results indicate that acoustic exposure significantly decreased the ABR thresholds of neonatal mice in a frequency-specific manner. The expression and function of prestin, the motor protein of OHCs, were specifically increased by acoustic stimulation. The number of ribbon synapses in the hair cell areas was also promoted by early acoustic stimulation.
